# Trends in and disparities for acute myocardial infarction: an analysis of Medicare claims data from 1992 to 2010

**DOI:** 10.1186/s12916-014-0190-6

**Published:** 2014-10-24

**Authors:** Jasvinder A Singh, Xin Lu, Said Ibrahim, Peter Cram

**Affiliations:** Medicine Service, Birmingham Veterans Affairs Medical Center, the University of Alabama at Birmingham, 510 S 20th Street, Faculty Office Tower 805B, Birmingham, AL 35294 UK; Division of General Internal Medicine, Department of Internal Medicine, University of Iowa Carver College of Medicine and CADRE, Iowa City Veterans Administration Medical Center, 451 Newton Road 200 Medicine Administration Building, Iowa City, IA 52242 USA; Center for Health Equity Research and Promotion, Philadelphia Veterans Affairs Medical Center, the Perelman University of Pennsylvania School of Medicine, 3400 Spruce St, Philadelphia, PA 19104 USA; Division of General Internal Medicine and Geriatrics, University Health Network and Mount Sinai Hospitals, 200 Elizabeth Street, Eaton North 14th Floor, Toronto, ON M5G 2C4 Canada; Faculty of Medicine, University of Toronto, Toronto, ON Canada

**Keywords:** Myocardial infarction, MI, Disparity, Outcomes, Race, Sex, Mortality, PCI, Hospitalization rates

## Abstract

**Background:**

It is unknown whether previously reported disparities for acute myocardial infarction (AMI) by race and sex have declined over time.

**Methods:**

We used Medicare Part A administrative data files for 1992 to 2010 to evaluate changes in per-capita hospitalization rates for AMI, rates of revascularization (percutaneous coronary intervention (PCI) and coronary artery bypass grafting (CABG)), and 30-day mortality for four distinct patient subcohorts: black women; black men; white women; and white men, adjusted for age, comorbidities and year using logistic regression.

**Results:**

The study sample consisted of 4,045,267 AMI admissions between the years 1992 and 2010 (166,660 black women; 116,201 black men; 1,870,816 white women; 1,891,590 white men). AMI hospitalization rates differed significantly in 1992 to 1993 among black women (61.6 hospitalizations per 10,000 Medicare enrollees), black men (73.2 hospitalizations), white women (72.0 hospitalizations) and white men (113.2 hospitalizations) (*P* <0.0001). By 2009 to 2010 AMI hospitalization rates had declined substantially in all cohorts but disparities remained with significantly lower hospitalization rates among women and blacks compared to men and whites, respectively (*P* <0.0001). In multivariable-adjusted analyses, despite narrowing of the differences between cohorts over time, disparities in AMI hospitalization rates by race and sex remained statistically significant in 2009 to 2010 (*P* <0.001). In 1992 to 1993 and 2009 to 2010, rates of PCI within 30-days of AMI differed significantly among black women (8.6% in 1992 to 1993; 24.2% in 2009 to 2010), black men (10.4% and 32.6%), white women (12.8% and 30.5%), and white men (16.1% and 40.7%) (*P* <0.0001). In multivariable-adjusted analyses, racial disparities in procedure utilization appeared somewhat larger and sex-based disparities remained significant. Unadjusted 30-day mortality after AMI in 1992 to 1993 for black women, black men, white women and white men was 20.4%, 17.9%, 23.1% and 19.5%, respectively (*P* <0.0001); in 2009 to 2010 mortality was 17.1%, 15.3%, 18.2% and 16.2%, respectively (*P* <0.0001). In adjusted analyses, racial differences in mortality declined over time but differences by sex (higher mortality for women) persisted.

**Conclusions:**

Disparities in AMI have declined modestly, but remain a problem, particularly with respect to patient sex.

**Electronic supplementary material:**

The online version of this article (doi:10.1186/s12916-014-0190-6) contains supplementary material, which is available to authorized users.

## Background

More than two decades of research has demonstrated significant disparities in the management of patients with acute myocardial infarction (AMI) based upon patient sex and race in both the United States (US) and Europe [1-5]. Reports from the US Institute of Medicine (IOM) and the European Union have called for providers and policymakers to confront and eliminate socioeconomic and racial and ethnic disparities in health care [6,7]. The World Health Organization has described the social determinants of health that lead to health inequities [8]. The US Affordable Care Act (ACA) established a dedicated office to reduce disparities [9].

While reduction of race- and sex-based disparities has been an international priority for decades, relatively few longitudinal studies have assessed the success of disparity reduction efforts. Moreover, most of the available studies were relatively short in duration (that is, 5 to 10 years) and few focused comprehensively on AMI [1,3]. In 2005, Jha *et al*. reported no decline in racial disparities for nine surgical procedures during the 1990s [10], but did not study disparities in AMI-associated hospitalization or mortality and this study has not been updated with contemporary data. The case fatality rate was consistently higher among Maori and Pacific Islands people than in Europeans in New Zealand for each age group and both sexes [5].

The overarching objective of our analysis was to comprehensively examine long-term trends in AMI disparities by patient race (black and white) and sex (male and female). In particular, we were interested in examining longitudinal differences in AMI-associated: 1) hospitalization rates; 2) utilization of revascularization procedures; and 3) mortality. In aggregate we felt that these three measures could comprehensively evaluate the full spectrum of AMI care from the underlying burden of cardiovascular disease (hospitalization rate), to processes of care (revascularization) and outcomes (mortality).

## Methods

### Data

We linked two US Medicare Provider Analysis and Review (MedPAR) Part A data files (one running from 1991 to 2005 and the second from 2006 to 2010) to identify fee-for-service beneficiaries hospitalized with AMI using International Classification of Diseases, Ninth Revision, Clinical Modification (ICD-9-CM) code, 410.xx. The MedPAR files contain administrative data for all fee-for-service Medicare enrollees hospitalized in US hospitals including: demographics (age, sex, race/ethnicity); ICD-9-CM codes for diagnoses and procedures; admission source (for example, emergency department or transfer from outside hospital); admission and discharge dates; discharge disposition (for example, home, nursing home, transfer to another acute-care hospital, dead); and death occurring up to three-years after discharge. The MedPAR files also include each patient’s unique Medicare beneficiary number allowing for tracking of patients transferred between hospitals.

We applied several inclusion and exclusion criteria [see Additional file 1] to generate our final analytic cohort. We began by identifying all Non-Hispanic white or black patients hospitalized with an AMI during our study period. We focused our analysis on blacks and whites because the black/white racial designation has traditionally been more accurate than other racial designations and the number of patients of ‘other’ race/ethnicity categories were relatively small, particularly during the early 1990s [11].

We identified a ‘new’ AMI by limiting our analysis to each patient’s first AMI hospitalization during a given one-year period by excluding index hospitalizations with prior AMI during the 12-months ‘look back’ preceding the index hospitalization. To implement the ‘look back’ we excluded patients <66 years of age at the time of their AMI and those lacking a full 12-months of MedPAR data preceding their AMI hospitalization; of necessity we excluded all patients hospitalized in 1991 and 2006 in order to allow us to apply the ‘look back’. We excluded all AMI admissions occurring in 2006 because a lack of consistent patient identification numbers across our two MedPAR files precluded us from linking patients across the two data sets. To be consistent with prior studies focusing on AMI [12-14], we excluded patients: 1) enrolled in Medicare HMOs; 2) with a primary diagnoses of 410.X2 (indicating the subsequent episode of care for AMI); 3) patients discharged alive within one day of admission not against medical advice, as such patients likely represent miscoding of AMI. We also excluded AMI admissions after 1 December 2005 for the 1991 to 2005 data file and after 1 December 2010 for the 2006 to 2010 data file to ensure a 30-day follow-up window for each admission.

### Study outcomes

We sought to evaluate changes in AMI disparities using an array of complementary measures, including hospitalization rates, receipt of revascularization procedures among those hospitalized and mortality [12-14]. Each measure was assessed in four discrete patient cohorts: black women, black men, white women, and white men. Revascularization included the receipt of three complementary cardiac procedures: 1) coronary angiography (with or without percutaneous coronary intervention (PCI); ICD-9-CM codes 37.21, 37.22, 37.23, 88.55, 88.56, 88.57); 2) PCI (ICD-9-CM codes 36.01, 36.02, 36.03, 36.04, 36.05, 36.06, 36.07,36.09, 00.66); and 3) coronary artery bypass surgery (CABG; ICD-9-CM codes 36.10, 36.11, 36.12, 36.13, 36.14, 36.15, 36.16, 36.17, 36.18, 36.19). We assessed receipt of each procedure at three different time-points: 1) during the index hospitalization (that is, at the admitting hospital); 2) during the index admission but after transfer from the admitting hospital to a second facility; and 3) within 30 days of AMI admission (which included procedures performed at the admitting hospital, after transfer, or during a subsequent readmission). Patients who underwent multiple procedures (for example, angiography plus CABG) were included in the counts for each. If a patient had more than one of the same procedure (for example, two PCIs within 30 days), we only counted the first procedure. We examined unadjusted and adjusted mortality within 30 days of AMI admission using methods described below.

### Statistical analysis

First, we compared the demographic characteristics and prevalence of key comorbid conditions by race and gender across the study period. We used analysis of variance (ANOVA) for comparisons of continuous variables and the chi-squared test for categorical variables. For simplicity, the study period was collapsed into two-year study increments (for example, 1992 to 1993, 1994 to 1995, and so on). Comorbid illnesses coded during the index admission were identified using algorithms developed by Elixhauser *et al*. [15].

Second, we compared unadjusted and standardized hospitalization rates for each of our four patient cohorts (black women, black men, white women and white men). AMI hospitalization rates were calculated as the number of AMI hospitalizations during a defined period of time in a given cohort (for example, 1992 to 1993) divided by the number of fee-for-service Medicare enrollees in that cohort during that period (for example, 1992 to 1993). We conducted comparisons across patient cohorts for a given time-period (for example, black women versus white women in 1992 to 1993) as well as longitudinal changes (for example, black women versus white women over the full study period) using statistical and graphical methods. We used analogous methods to compare differences in the utilization of cardiac procedures (catheterization, PCI and CABG) across patient cohorts (for example, black women compared to white women during a fixed time-period (1992 to 1993)) as well as to compare longitudinal changes over time.

Third, we examined trends in unadjusted and risk-adjusted 30-day mortality using hierarchical generalized linear models. Our models were based upon models developed by the US Centers for Medicare and Medicaid Services and adjusted for patient demographics and comorbidities and accounted for clustering of patients within hospitals [16]. For 30-day mortality, rates were adjusted for age, year, location of MI, unstable angina, chronic atherosclerosis, cardiopulmonary-respiratory failure and shock, valvular heart disease, hypertension, stroke, cerebrovascular disease, renal failure, COPD, pneumonia, diabetes, protein-calorie malnutrition, dementia, hemiplegia, paraplegia, paralysis, functional disability, peripheral vascular disease, metastatic cancer, trauma in last year, major psychiatric disorders, chronic liver disease, history of PCI, history of CABG and history of heart failure.

Finally, we conducted an array of sensitivity analyses. First, we repeated our analyses of procedure utilization using an endpoint of 30-day revascularization (PCI or CABG) in place of our focus on individual procedures. Second, we repeated our evaluations of procedure utilization using models that adjusted for patient age and comorbidities and accounted for clustering of patients within hospitals; these models allowed us to examine whether differences in utilization might be explained by differences in patient comorbidity and/or differences in the hospitals where each patient cohort received care. Third, we repeated our analyses without applying the one-year look-back, which allowed us to include patients from 1991 and 2006 in our analyses. Fourth, in order to explore differences in the timing of revascularization, we examined the proportion of patients who received a procedure during the index hospitalization, after transfer to another acute care hospital, and during a separate hospital admission but within 30-days of the index admission in each of our four patient cohorts. All sensitivity analyses and model details are available by request. All analyses were performed using SAS Version 9.2 (Cary, NC, USA). The University of Iowa Institutional Review Board approved this study.

## Results

### Cohort characteristics

Our study consisted of 4,045,267 AMI admissions between 1992 and 2010 [see Additional file 1] of which 166,660 admissions were black women, 116,201 black men, 1,870,816 white women and 1,891,590 were white men. Black and white women hospitalized with AMI were consistently older than black and white men (Table 1). Among each of the four patient cohorts, age increased by approximately three-years over the study period (Table 1).

**Table 1:** **Characteristics of black and white men and women hospitalized with acute myocardial infarction (AMI)**

	**Black women**	**Black men**	**White women**	**White men**
**Hospitalizations, number (rate per 10,000 Medicare enrollees)** ^**a**^				
1992-1993	16237 (61.6)	11958 (73.2)	219273 (72.0)	228751 (113.2)
1994-1995	17774 (63.4)	12889 (75.6)	227347 (72.4)	237520 (112.9)
1996-1997	18541 (64.9)	13351 (77.3)	228918 (72.5)	235828 (110.3)
1998-1999	19562 (67.3)	13167 (75.0)	226734 (71.5)	226984 (104.5)
2000-2001	21100 (70.5)	14091 (77.5)	231992 (72.6)	226802 (102.4)
2002-2003	21918 (71.7)	14595 (77.8)	228463 (71.5)	223957 (99.3)
2004-2005^b^	18276 (59.3)	12743 (65.7)	190021 (59.9)	187818 (81.5)
2007-2008	17043 (49.3)	11961 (54.3)	169446 (49.9)	170004 (67.2)
2009-2010	16209 (43.1)	11446 (46.7)	148622 (41.0)	153926 (55.6)
**Age, mean (standard deviation)**				
1992-1993	77.1 (7.6)	75.1 (6.8)	78.4 (7.4)	75.5 (6.7)
1994-1995	77.3 (7.7)	75.3 (6.9)	78.7 (7.5)	75.8 (6.8)
1996-1997	77.8 (7.8)	75.7 (7.1)	79.4 (7.6)	76.4 (7.0)
1998-1999	78.6 (8.0)	76.1 (7.2)	80.1 (7.7)	77.1 (7.1)
2000-2001	79.0 (8.2)	76.5 (7.4)	80.6 (7.8)	77.5 (7.3)
2002-2003	79.2 (8.3)	76.4 (7.5)	80.9 (7.9)	77.7 (7.4)
2004-2005	79.7 (8.3)	76.6 (7.5)	81.2 (8.0)	78.1 (7.5)
2007-2008	79.8 (8.7)	76.5 (7.7)	81.5 (8.3)	78.1 (7.9)
2009-2010	79.7 (8.7)	76.3 (7.8)	81.6 (8.4)	78.1 (8.1)
**Comorbidity, number (%)** ^**c**^				
**Diabetes**				
1992-1993	5671 (34.9)	2855 (23.9)	50955 (23.2)	44725 (19.6)
1994-1995	6975 (39.2)	3648 (28.3)	58780 (25.9)	53720 (22.6)
1996-1997	7700 (41.5)	4004 (30.0)	60433 (26.4)	56631 (24.0)
1998-1999	7993 (40.9)	4146 (31.5)	60627 (26.7)	57138 (25.2)
2000-2001	8639 (40.9)	4608 (33.7)	61893 (26.7)	59138 (26.1)
2002-2003	8646 (39.5)	4815 (33.0)	59646 (26.1)	58803 (26.3)
2004-2005	7021 (38.4)	4171 (32.7)	48017 (25.3)	48774 (26.0)
2007-2008	6262 (36.7)	3791 (31.7)	40832 (24.1)	43512 (25.6)
2009-2010	5867 (36.2)	3589 (31.4)	35738 (24.1)	39389 (25.6)
**Congestive heart failure, number (%)** ^**c**^				
1992-1993	7662 (47.2)	4895 (40.9)	98207 (44.8)	86088 (37.6)
1994-1995	8433 (47.4)	5403 (41.9)	103382 (45.5)	89728 (37.8)
1996-1997	9149 (49.3)	5928 (44.4)	105829 (46.2)	91840 (38.9)
1998-1999	9883 (50.5)	5897 (44.8)	106226 (46.8)	89515 (39.4)
2000-2001	10501 (49.8)	6335 (45.0)	107129 (46.2)	88753 (39.1)
2002-2003	10892 (49.7)	6565 (45.0)	107208 (46.9)	89274 (39.9)
2004-2005	9375 (51.3)	5775 (45.3)	91500 (48.2)	77046 (41.0)
2007-2008	7989 (46.9)	5189 (43.4)	75421 (44.5)	63959 (37.6)
2009-2010	7439 (45.9)	4817 (42.1)	64552 (43.4)	56955 (37.0)
**Obesity, number (%)** ^**c**^				
1992-1993	375 (2.3)	101 (0.8)	3888 (1.8)	2752 (1.2)
1994-1995	609 (3.4)	176 (1.4)	5458 (2.4)	3899 (1.6)
1996-1997	696 (3.8)	190 (1.4)	6194 (2.7)	4695 (2.0)
1998-1999	701 (3.6)	227 (1.7)	6172 (2.7)	4873 (2.2)
2000-2001	825 (3.9)	257 (1.8)	6540 (2.8)	5322 (2.4)
2002-2003	954 (4.4)	298 (2.0)	6528 (2.9)	5820 (2.6)
2004-2005	834 (4.6)	286 (2.2)	5860 (3.1)	5410 (2.9)
2007-2008	819 (4.8)	313 (2.6)	5478 (3.2)	5802 (3.4)
2009-2010	864 (5.3)	353 (3.1)	5329 (3.6)	6009 (3.9)

^a^The denominator is all traditional Medicare enrollees in each stratum, that is, black female, black male, white female, and white male; ^b^patients from calendar year 2006 were excluded as detailed in the Methods section; ^c^The denominator is AMI patients in each stratum, that is, black female, black male, white female, and white male.

### AMI hospitalization rates

**Figure 1:**
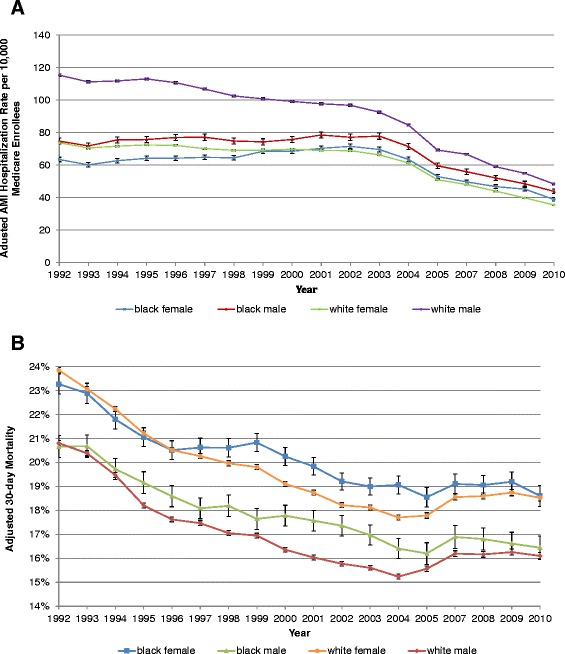
**Adjusted hospitalization rates for AMI (A) and 30-day all-cause mortality after admission for AMI (B).** Y-axis shows adjusted AMI hospitalization per 10,000 Medicare enrollees; rates were adjusted for age and year **(A)**. For 30-day all-cause mortality, rates were adjusted for age, year, location of MI, unstable angina, chronic atherosclerosis, cardiopulmonary-respiratory failure and shock, valvular heart disease, hypertension, stroke, cerebrovascular disease, renal failure, COPD, pneumonia, diabetes, protein-calorie malnutrition, dementia, hemiplegia, paraplegia, paralysis, functional disability, peripheral vascular disease, metastatic cancer, trauma in last year, major psychiatric disorders, chronic liver disease, history of PCI, history of CABG and history of heart failure **(B)**. AMI, acute myocardial infarction; CABG, coronary artery bypass surgery; COPD, chronic obstructive pulmonary disease; PCI, percutaneous coronary intervention.

The per-capita hospitalization rate for AMI (number of hospitalizations during a specific year/number of Medicare enrollees in that specific patient group) differed markedly across the four study cohorts (Table 1 and Figure 1A), although there was evidence that the disparities narrowed over time. In particular during the early 1990s, white men had a higher hospitalization rate and white women a lower hospitalization rate (*P* <0.001). Despite narrowing of the differences between cohorts over time, disparities in AMI hospitalization rates by race and sex remained statistically significant in 2009 to 2010 (*P* <0.001). Viewed from an alternate perspective, each cohort experienced significant decreases in AMI hospitalization rates between 1992 to 1993 and 2009 to 2010 (*P* <0.01 for each cohort over time).

### Procedure utilization

**Figure 2:**
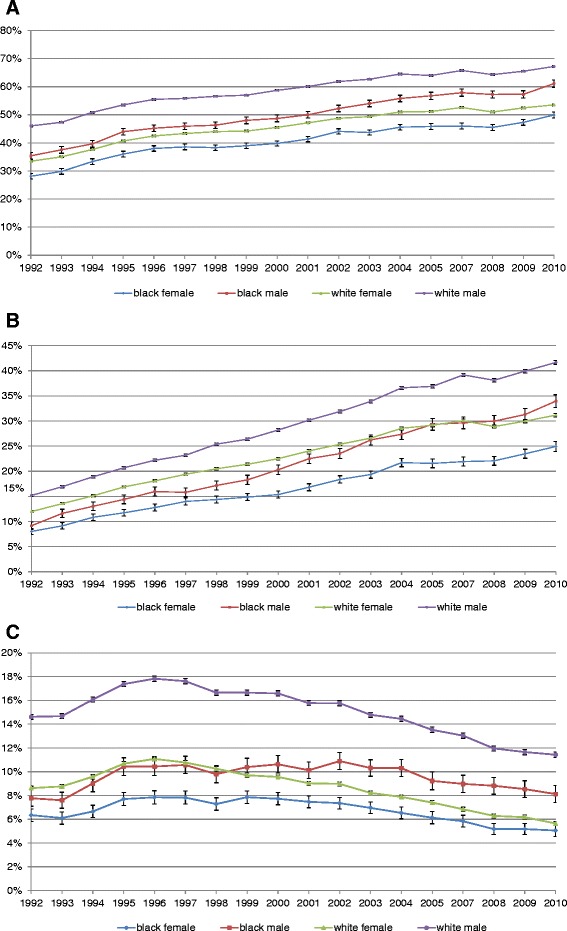
**Unadjusted utilization of coronary angiography (A), PCI (B) and CABG (C) within 30 days after admission for AMI.** Y-axis shows the unadjusted percentage of Medicare beneficiaries hospitalized with AMI receiving angiography (with or without revascularization) **(A)**, PCI **(B)** or CABG **(C)** within 30 days of admission. AMI, acute myocardial infarction; CABG, coronary artery bypass surgery; PCI, percutaneous coronary intervention.

Unadjusted rates of all procedures (coronary angiography, PCI and CABG) within 30 days of AMI admission were lower for blacks compared to whites and women compared to men throughout the study period (Table 2 and Figures 2A, B, and C). For example, in 1992 to 1993 29.0% of black women and 36.5% of black men underwent coronary angiography (with or without PCI) within 30 days of AMI admission as compared to 34.3% of white women and 46.7% of white men (*P* <0.0001). Likewise, in 2009 to 2010, 5.1% of black women and 8.3% of black men received CABG within 30 days of AMI admission compared to 5.9% of white women and 11.6% of white men (*P* <0.0001). Viewed longitudinally, all patient cohorts saw an approximately 20% rise in the percentage of patients who received coronary angiography within 30 days of AMI admission over the study period (Table 2 and Figures 2A, B, and C). In addition, the proportion of these procedures that were performed during the index admission increased over time for all patient cohorts [see Additional files 2, 3 and 4].

**Table 2:** **Percentage of black and white men and women who underwent selected procedures within 30 days of admission for AMI**

	**Black women**	**Black men**	**White women**	**White men**
**Coronary angiography, number (%)**				
**Number (%) who underwent the procedure within 30 days of admission** ^**a**^				
1992-1993	4707 (29.0)	4362 (36.5)	75134 (34.3)	106829 (46.7)
1994-1995	6169 (34.7)	5391 (41.8)	89097 (39.2)	123998 (52.2)
1996-1997	7101 (38.3)	6086 (45.6)	98228 (42.9)	131268 (55.7)
1998-1999	7563 (38.7)	6209 (47.2)	99993 (44.1)	128880 (56.8)
2000-2001	8565 (40.6)	6955 (49.4)	107484 (46.3)	134706 (59.4)
2002-2003	9623 (43.9)	7757 (53.2)	112046 (49.0)	139416 (62.3)
2004-2005	8356 (45.7)	7168 (56.3)	97082 (51.1)	120720 (64.3)
2007-2008	7799 (45.8)	6883 (57.6)	87771 (51.8)	110588 (65.1)
2009-2010	7867 (48.5)	6768 (59.1)	78727 (53.0)	102085 (66.3)
**Among those who underwent angiography, number (%) who received their angiography during the index admission** ^**b**^				
1992-1993	3456 (73.4)	3193 (73.2)	46938 (62.5)	68346 (64.0)
1994-1995	4544 (73.7)	4013 (74.4)	55723 (62.5)	80312 (64.8)
1996-1997	5260 (74.1)	4500 (73.9)	63176 (64.3)	86863 (66.2)
1998-1999	5560 (73.5)	4674 (75.3)	66205 (66.2)	87498 (67.9)
2000-2001	6486 (75.7)	5245 (75.4)	73240 (68.1)	94081 (69.8)
2002-2003	7465 (77.6)	6074 (78.3)	80892 (72.2)	103264 (74.1)
2004-2005	6759 (80.9)	5869 (81.9)	74730 (77.0)	95221 (78.9)
2007-2008	6521 (83.6)	5826 (84.6)	72270 (82.3)	93147 (84.2)
2009-2010	6791 (86.3)	5887 (87.0)	66669 (84.7)	88275 (86.5)
**Percutaneous coronary intervention (PCI), number (%)**				
**Number (%) who underwent the procedure within 30 days of index AMI admission** ^**a**^				
1992-1993	1394 (8.6)	1243 (10.4)	28013 (12.8)	36743 (16.1)
1994-1995	2009 (11.3)	1768 (13.7)	36406 (16.0)	47057 (19.8)
1996-1997	2483 (13.4)	2120 (15.9)	42885 (18.7)	53486 (22.7)
1998-1999	2861 (14.6)	2335 (17.7)	47506 (21.0)	58746 (25.9)
2000-2001	3398 (16.1)	3019 (21.4)	53981 (23.3)	66189 (29.2)
2002-2003	4134 (18.9)	3633 (24.9)	59331 (26.0)	73620 (32.9)
2004-2005	3956 (21.7)	3602 (28.3)	54776 (28.8)	68973 (36.7)
2007-2008	3748 (22.0)	3564 (29.8)	49938 (29.5)	65672 (38.6)
2009-2010	3916 (24.2)	3728 (32.6)	45375 (30.5)	62705 (40.7)
**Among those who underwent PCI, number (%) during the index admission** ^**b**^				
1992-1993	857 (61.5)	754 (60.7)	14567 (52.0)	19915 (54.2)
1994-1995	1241 (61.8)	1120 (63.4)	19519 (53.6)	26582 (56.5)
1996-1997	1600 (64.4)	1329 (62.7)	24381 (56.9)	31498 (58.9)
1998-1999	1841 (64.4)	1497 (64.1)	28156 (59.3)	35960 (61.2)
2000-2001	2330 (68.6)	2026 (67.1)	33527 (62.1)	42653 (64.4)
2002-2003	2916 (70.5)	2607 (71.8)	40308 (67.9)	51497 (70.0)
2004-2005	2972 (75.1)	2802 (77.8)	40402 (73.8)	52304 (75.8)
2007-2008	2999 (80.0)	2926 (82.1)	40387 (80.9)	54283 (82.7)
2009-2010	3315 (84.7)	3148 (84.4)	38285 (84.4)	53819 (85.8)
**Coronary artery bypass grafting (CABG), number (%)**				
**Number (%) who underwent the procedure within 30 days of index AMI admission** ^**a**^				
1992-1993	1011 (6.2)	919 (7.7)	19058 (8.7)	33508 (14.7)
1994-1995	1277 (7.2)	1254 (9.7)	23047 (10.1)	39739 (16.7)
1996-1997	1453 (7.8)	1402 (10.5)	25006 (10.9)	41789 (17.7)
1998-1999	1483 (7.6)	1329 (10.1)	22626 (10.0)	37796 (16.7)
2000-2001	1603 (7.6)	1461 (10.4)	21567 (9.3)	36690 (16.2)
2002-2003	1569 (7.2)	1547 (10.6)	19654 (8.6)	34226 (15.3)
2004-2005	1160 (6.4)	1250 (9.8)	14560 (7.7)	26342 (14.0)
2007-2008	937 (5.5)	1064 (8.9)	11125 (6.6)	21249 (12.5)
2009-2010	830 (5.1)	954 (8.3)	8806 (5.9)	17773 (11.6)
**Among those who underwent CABG, number (%) during the index admission** ^**b**^				
1992-1993	519 (51.3)	435 (47.3)	7747 (40.7)	14152 (42.2)
1994-1995	624 (48.9)	647 (51.6)	9537 (41.4)	17294 (43.5)
1996-1997	713 (49.1)	729 (52.0)	10868 (43.5)	19127 (45.8)
1998-1999	766 (51.7)	703 (52.9)	10541 (46.6)	18400 (48.7)
2000-2001	871 (54.3)	770 (52.7)	10617 (49.2)	18859 (51.4)
2002-2003	913 (58.2)	905 (58.5)	10726 (54.6)	19471 (56.9)
2004-2005	704 (60.7)	787 (63.0)	8822 (60.6)	16693 (63.4)
2007-2008	604 (64.5)	697 (65.5)	7369 (66.2)	14595 (68.7)
2009-2010	565 (68.1)	648 (67.9)	5883 (66.8)	12332 (69.4)
**Revascularization (CABG or PCI), number (%)**				
**Number (%) who underwent the procedure within 30 days of index AMI admission** ^**a**^				
1992-1993	2325 (14.3)	2108 (17.6)	45598 (20.8)	67970 (29.7)
1994-1995	3211 (18.1)	2943 (22.8)	57580 (25.3)	84056 (35.4)
1996-1997	3864 (20.8)	3454 (25.9)	66268 (29.0)	92920 (39.4)
1998-1999	4269 (21.8)	3604 (27.4)	68755 (30.3)	94321 (41.6)
2000-2001	4921 (23.3)	4413 (31.3)	74229 (32.0)	100637 (44.4)
2002-2003	5606 (25.6)	5080 (34.8)	77563 (34.0)	105419 (47.1)
2004-2005	5028 (27.5)	4758 (37.3)	68167 (35.9)	93067 (49.6)
2007-2008	4598 (27.0)	4540 (38.0)	59979 (35.4)	84696 (49.8)
2009-2010	4685 (28.9)	4605 (40.2)	53326 (35.9)	78561 (51.0)
**Among those who underwent revascularization, number (%) during the index admission** ^**b**^				
1992-1993	1336 (57.5)	1157 (54.9)	21524 (47.2)	32793 (48.3)
1994-1995	1820 (56.7)	1712 (58.2)	28006 (48.6)	42250 (50.3)
1996-1997	2268 (58.7)	2014 (58.3)	34286 (51.7)	49217 (53.0)
1998-1999	2570 (60.2)	2163 (60.0)	37872 (55.1)	53006 (56.2)
2000-2001	3156 (64.1)	2754 (62.4)	43410 (58.5)	60168 (59.8)
2002-2003	3769 (67.2)	3446 (67.8)	50186 (64.7)	69457 (65.9)
2004-2005	3630 (72.2)	3522 (74.0)	48465 (71.1)	67455 (72.5)
2007-2008	3541 (77.0)	3568 (78.6)	47008 (78.4)	67286 (79.4)
2009-2010	3837 (81.9)	3741 (81.2)	43575 (81.7)	64784 (82.5)

^a^The denominator is AMI patients in each stratum, that is, black women, black men, white women and white men; ^b^the denominator is AMI patients who underwent the respective procedure in each stratum, that is, black women, black men, white women and white men. AMI, acute myocardial infarction; CABG, coronary artery bypass surgery; PCI, percutaneous coronary intervention.

In analyses that adjusted for patient age and comorbidity [see Additional file 5] and additionally accounting for clustering of patients within hospitals [see Additional file 6], results were somewhat different. In particular, we found that the lower procedure utilization in blacks as compared to whites was largely eliminated after accounting for differences in age and comorbidity [see Additional file 5] although disparities by sex (lower procedure utilization among women as compared to men) persisted. In analyses that also accounted for clustering of patients within hospitals, racial disparities in procedure utilization appeared somewhat larger and sex-based disparities remained significant [see Additional file 6].

### Mortality

Unadjusted mortality within 30 days of admission was lower for blacks than for whites and for men than for women throughout the study period (Table 3), although differences appeared to decline over time. For example, in 1992 to 1993, the 30-day mortality rate after index AMI was lower in black men compared to white men (17.9% versus 19.5%; *P* ≤0.0001) and in black women compared to white women (20.4% versus 23.1%; *P* <0.0001). By 2009 to 2010, absolute differences between groups had narrowed, but remained significant (Table 3). In analyses that adjusted for differences in demographics and comorbidity (Figure 1B), racial differences in mortality declined over time but differences by sex (higher mortality for women) persisted. Viewed from a different perspective 30-day mortality after AMI decreased significantly (*P* <0.0001) within each cohort over the study period.

**Table 3:** **Percentage** ^**a**^ **of black and white men and women who died within 30 days after admission for AMI (unadjusted estimates)**

	**Black women**	**Black men**	**White women**	**White male**
**Mortality, number (%)**				
1992-1993	3311 (20.4)	2145 (17.9)	50641 (23.1)	44578 (19.5)
1994-1995	3492 (19.7)	2189 (17.0)	50269 (22.1)	43655 (18.4)
1996-1997	3512 (18.9)	2139 (16.0)	48151 (21.0)	41907 (17.8)
1998-1999	3761 (19.2)	2213 (16.8)	46539 (20.5)	40065 (17.7)
2000-2001	3952 (18.7)	2361 (16.8)	45521 (19.6)	38349 (16.9)
2002-2003	3847 (17.6)	2396 (16.4)	42912 (18.8)	36805 (16.4)
2004-2005	3122 (17.1)	1921 (15.1)	34193 (18.0)	29289 (15.6)
2007-2008	2751 (16.1)	1790 (15.0)	29842 (17.6)	26226 (15.4)
2009-2010	2768 (17.1)	1751 (15.3)	26994 (18.2)	24889 (16.2)

^a^The denominator is number of AMI patients in each stratum (that is, black women, black men, white women and white men) during each two-year period. AMI, acute myocardial infarction.

## Discussion

In a longitudinal analysis of US Medicare beneficiaries hospitalized with AMI between 1992 and 2010, we report a number of important trends in disparities. We found that AMI hospitalization rates declined substantially for all patient groups. We also found that differences in hospitalization rates in blacks and whites and men and women have narrowed over time, but disparities persist. Utilization of interventional procedures after AMI has increased over time, but disparities persist with both women and blacks being significantly less likely to receive most procedures. Lastly, 30-day mortality following an AMI has declined over time for all patient groups, but remains significantly higher for women compared to men. Several findings merit further discussion.

First, we found declines in overall hospitalization rates for AMI among all four of our study cohorts (black women, black men, white women, and white men). While there are several studies reporting changes over time in AMI mortality or quality of AMI care [17-19], there are fewer studies of long-term trends in AMI hospitalization rates, with somewhat contradictory results. Chen and Krumholz examined trends in AMI hospitalization among Medicare beneficiaries between 2002 and 2007 and noted an approximately 20% decline in hospitalization rates [20]; they also found evidence of higher AMI hospitalization rates among men compared to women and white men as compared to black men. Similarly, Talbott *et al*. reported a 20% decline in AMI hospitalization rates using the Centers for Disease Control (CDC) data [21]. Alternatively, data show declines in cardiovascular disease mortality over time without corresponding reductions in AMI hospitalization rates [22]. Our findings are particularly intriguing when viewed through the lens of increasingly sensitive diagnostic tests for AMI (that is, serum troponin) that would be expected to increase AMI diagnosis and hospitalization rates [23]. In a nationwide Danish study, the standardized incidence rate per 100,000 people decreased in the 25-year period, 1984 to 2008, by 37% for women (from 209 to 131) and by 48% for men (from 410 to 213) [24].

Our finding of differential hospitalization rates by race and sex is noteworthy and merits brief comment. It is possible that the lower hospitalization rates truly represent lower population-based AMI risk among blacks (compared to whites) and women (compared to men). However, an array of epidemiological data suggests a higher burden of cardiovascular disease among blacks making this scenario unlikely [25,26]. An alternative and more ominous explanation is that the lower hospitalization rates reflect under-diagnosis of AMI in blacks and women in the pre-hospital setting. Differences in AMI hospitalization rates by race and sex remained statistically significant in 2009 to 2010 (*P* <0.001) despite narrowing of the differences between cohorts over time in analyses adjusted for age and year (Figure 1A). This implies that only some racial and sex differences were attributable to age and period effects, and some disparities still persisted, although they narrowed in adjusted analyses. Another study using the CDC data reported that the male–female ratio for AMI hospitalization rates remained constant at 2:1 from 2000 to 2008 [21].

Second, we found a marked increase in the utilization of revascularization among all patient cohorts over time. These results are consistent with prior studies [22]. Interestingly, while disparities in revascularization have been well documented [27], relatively few studies have examined whether disparities in revascularization have declined over time [28]. Peterson *et al*., using data from the National Registry of Myocardial Infarction (NRMI) found evidence that disparities by race and sex in the use of revascularization procedures did not decline between 1990 and 2006 [18]. Alternatively, Jha and colleagues found evidence that disparities for certain revascularization procedures declined among certain patient populations between 1992 and 2001 (for example, PCI in black and white women) but other disparities persisted (for example, CABG in black and white men) [10]. Our results extend prior studies by examining a more comprehensive list of revascularization procedures over a longer time period. Our unadjusted analyses showed minimal reductions in disparities for most procedures and that disparities between men and women remain particularly large. Interestingly, in analyses that adjusted for patient comorbidity and clustering of patients within hospitals [see Additional file 1], racial disparities appeared small although disparities by patient sex remained significant. The differences between our unadjusted and adjusted results are consistent with prior studies that have suggested that many disparities can be explained by differences in patients’ acuity and comorbidity and also the quality and practice patterns of the physicians and hospitals where different patient groups seek care [2,18,29,30].

Third, our findings with regards to mortality are important and extend prior work. We found that racial disparities in AMI 30-day mortality grew relatively larger in the early part of the 21^st^ century, but have declined over the last five years. Alternatively, disparities by patient sex (higher mortality for both black and white women when compared to their male counterparts) have declined little [31]; higher mortality has been reported in younger (<50 years), but not older women compared to men of the same age after AMI [32]. Studies have suggested that sex-based disparities in mortality can be attributed in part to differences in the receipt of evidence-based treatments and interventions, but also perhaps to differences in AMI presentation, severity and age-of-onset [33]. Irrespective of the exact cause, the sex-based differences in AMI mortality are striking, similar to a previous population-based Finnish study [34]. In this study including all age groups comparing 1994 to 1996 to 2000 to 2002 in two registers, the 28-day case fatality after AMI declined in men from 46.5% to 41.0% in the FINAMI register and from 54.7% to 50.1% in the Finnish National Cardiovascular Disease Register (CVDR), respectively. The corresponding numbers in women decreased from 53.5% to 48.6% in FINAMI and from 58.9% to 54.6% in CVDR [34]. Another recent study found that mortality from ischemic heart disease increased across all ages in adult women whereas the corresponding increase was blunted at older ages in men [35], a potential explanation of sex differences in AMI mortality in our data as well. A Dutch study of 14,434 patients in coronary units from 1985 to 2008 showed no sex difference in adjusted 30-day mortality [36], as well as a decline in mortality in both men and women over a 24-year period. The differences in country setting, health care delivery system, patient comorbidity load and single center versus national data between this previous study and our study may explain the differences in findings. Our mortality models were based on models developed by the US Centers for Medicare and Medicaid Services and adjusted for hospital clustering and, therefore, our confidence in these findings is high.

Our study has several limitations that warrant mention. First, our analysis was limited to fee-for-service Medicare beneficiaries and should be extended to other populations with caution. In particular, it is uncertain whether an evaluation of a younger non-Medicare population would yield similar results. Second, our analysis focused exclusively on AMI and we cannot comment on long-term trends in disparities for other conditions. Third, our analyses could have been impacted by changes in coding over time related to either AMI diagnosis or procedures. However, there is a long history of using Medicare data in the evaluation of AMI and we are unaware of any coding changes that would have directly impacted our results. Fourth, Medicare data may have race misclassification since this was not self-identified prospectively; however, this would bias our findings towards null and, therefore, our findings are conservative. In addition, this is a general limitation of studies using Medicare data. The impact of the exclusion of 2006 is unclear; however we have a long-enough observation period, and a single year is unlikely to have meaningfully changed the findings. Fifth, the time-trends analyses for where the procedures were done are unadjusted (index hospitalization, transfer hospitalization, or post-discharge within 30 days); it is anticipated that a reduction in the length of AMI stay over the study period likely impacted these rates, and, therefore, these should be interpreted with caution. Lastly, we are unable to account for patient preferences, which play a role in utilization of these procedures or physician preferences, or the clinical information about the appropriateness of the interventional procedures. This is an important aspect of disparities that needs to be understood; however, this is likely on the causal pathway and, therefore, not a confounder of the estimates presented in this study.

## Conclusions

In summary, over the past 18 years we observed declines in hospitalization rates and mortality and increases in revascularization for AMI in the US Medicare population. We found that disparities by sex and also by race in hospitalization rates, receipt of revascularization procedures and mortality have persisted over time. Our study provides evidence of the continued challenge of insuring equal care to all patient groups.

## Additional files

Additional file 1:
**Flow Chart showing patient selection for AMI cohort for our study.**
Additional file 2:
**Percentage of White and Black Men and Women who underwent Coronary Angiography within 30-day of admission for AMI.**
Additional file 3:
**Percentage of White and Black Men and Women who underwent percutaneous coronary intervention (PCI) within 30-day of admission for AMI.**
Additional file 4:
**Percentage of White and Black Men and Women who underwent Coronary Artery Bypass Graft (CABG) within 30-day after admission for AMI.**
Additional file 5:
**Procedural Utilization During the Index Admission for initial AMI Adjusted for Patient Age and Comorbidities.**
Additional file 6:
**Procedural Utilization During the Index Admission for initial AMI Adjusted for Patient Age and Comorbidities, and Clustering of Patients within Hospitals.**
