# Supplementary figures and images for: Trends in and disparities for acute myocardial infarction: an analysis of Medicare claims data from 1992 to 2010
Source: BMC Med. 2014 Oct 24;12:190. doi: 10.1186/s12916-014-0190-6 (PMC4212130; doi:10.1186/s12916-014-0190-6)

**Additional file 1.** Flow Chart showing patient selection for AMI cohort for our study

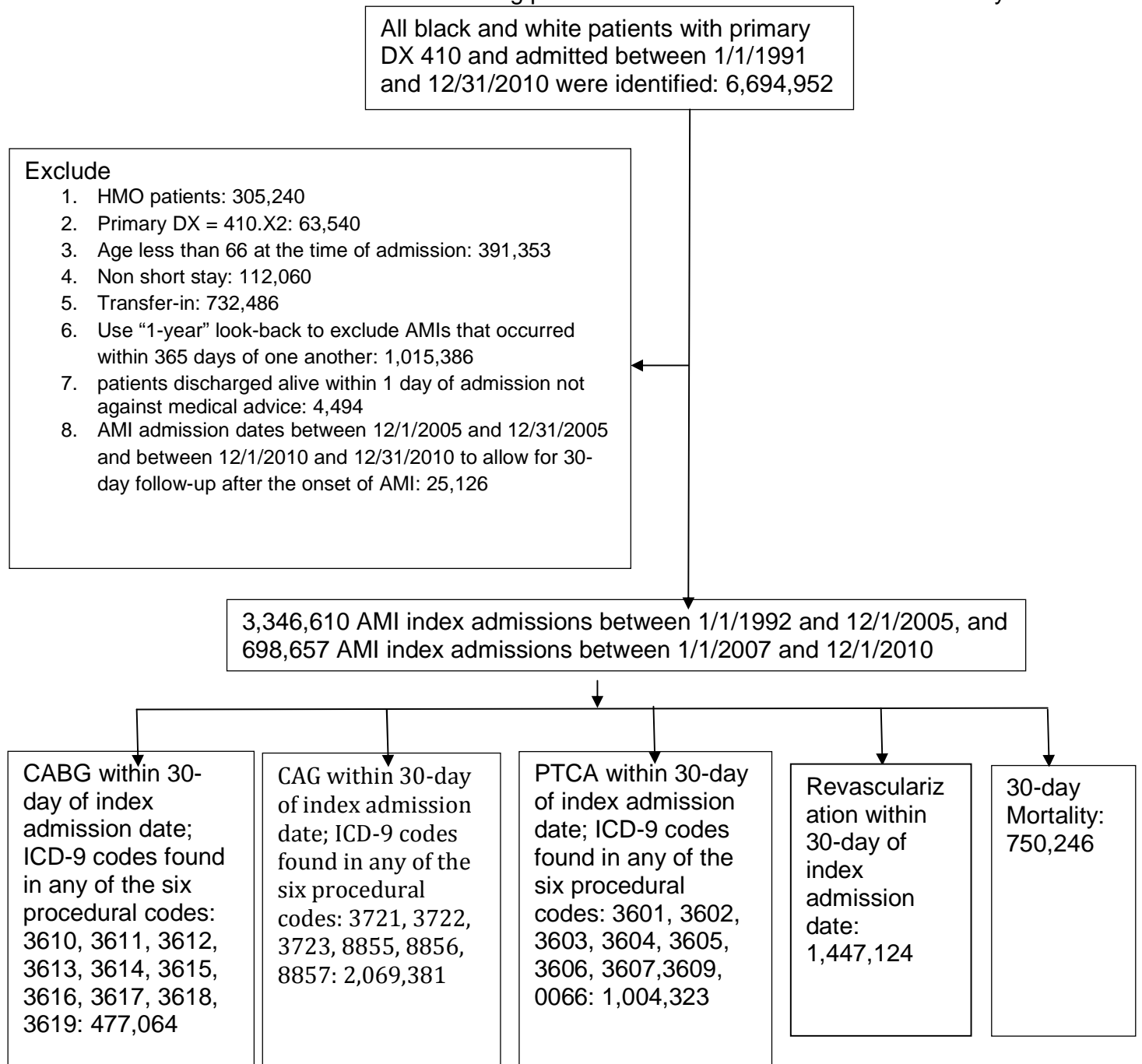

Supplement: Additional file 1: — Flow Chart showing patient selection for AMI cohort for our study. [file 12916_2014_190_MOESM1_ESM.pdf]
