# Supplementary material for: Trends in and disparities for acute myocardial infarction: an analysis of Medicare claims data from 1992 to 2010
Source: BMC Med. 2014 Oct 24;12:190. doi: 10.1186/s12916-014-0190-6 (PMC4212130; doi:10.1186/s12916-014-0190-6)
Supplement: Additional file 2: — Percentage of White and Black Men and Women who underwent Coronary Angiography within 30-day of admission for AMI. [file 12916_2014_190_MOESM2_ESM.pdf]

Additional file 2. Percentage of White and Black Men and Women who underwent Coronary Angiography within 30-day of admission for AMI

	**BLACK FEMALE**	**BLACK MALE**	**WHITE FEMALE**	**WHITE MALE**
**Number (%)**				
**Percent of all Coronary Angiography in 30-day period that were performed during the initial AMI admission[2]**				
1992-1993	3456 (73.4)	3193 (73.2)	46938 (62.5)	68346 (64.0)
1994-1995	4544 (73.7)	4013 (74.4)	55723 (62.5)	80312 (64.8)
1996-1997	5260 (74.1)	4500 (73.9)	63176 (64.3)	86863 (66.2)
1998-1999	5560 (73.5)	4674 (75.3)	66205 (66.2)	87498 (67.9)
2000-2001	6486 (75.7)	5245 (75.4)	73240 (68.1)	94081 (69.8)
2002-2003	7465 (77.6)	6074 (78.3)	80892 (72.2)	103264 (74.1)
2004-2005	6759 (80.9)	5869 (81.9)	74730 (77.0)	95221 (78.9)
2007-2008	6521 (83.6)	5826 (84.6)	72270 (82.3)	93147 (84.2)
2009-2010	6791 (86.3)	5887 (87.0)	66669 (84.7)	88275 (86.5)
**Percent of all Coronary Angiography in 30-day period that were performed after transfer to another hospital[2]**				
1992-1993	965 (20.5)	917 (21.0)	22790 (30.3)	31209 (29.2)
1994-1995	1267 (20.5)	1114 (20.7)	27373 (30.7)	36084 (29.1)
1996-1997	1448 (20.4)	1278 (21.0)	28882 (29.4)	36784 (28.0)
1998-1999	1586 (21.0)	1231 (19.8)	27720 (27.7)	34244 (26.6)
2000-2001	1662 (19.4)	1346 (19.4)	28168 (26.2)	33698 (25.0)
2002-2003	1719 (17.9)	1335 (17.2)	25631 (22.9)	29906 (21.5)
2004-2005	1249 (15.0)	996 (13.9)	18115 (18.7)	20508 (17.0)
2007-2008	980 (12.6)	783 (11.4)	11981 (13.7)	13193 (11.9)
2009-2010	804 (10.2)	612 (9.0)	9070 (11.5)	10254 (10.0)
**Percent of all Coronary Angiography in 30-day period that were performed during a different admission (but within 30-day of the onset of AMI)[2]**				
1992-1993	286 (6.1)	252 (5.8)	5406 (7.2)	7274 (6.8)
1994-1995	358 (5.8)	264 (4.9)	6001 (6.7)	7602 (6.1)
1996-1997	393 (5.5)	308 (5.1)	6170 (6.3)	7621 (5.8)
1998-1999	417 (5.5)	304 (4.9)	6068 (6.1)	7138 (5.5)
2000-2001	417 (4.9)	364 (5.2)	6076 (5.7)	6927 (5.1)
2002-2003	439 (4.6)	348 (4.5)	5523 (4.9)	6246 (4.5)
2004-2005	348 (4.2)	303 (4.2)	4237 (4.4)	4991 (4.1)
2007-2008	298 (3.8)	274 (4.0)	3520 (4.0)	4248 (3.8)
2009-2010	272 (3.5)	269 (4.0)	2988 (3.8)	3556 (3.5)

[1] The denominator is AMI patients in each strata, i.e., black female, black male, white female, and white male.

[2] The denominator is AMI patients who underwent **coronary angiography** in each strata, i.e., black female, black male, white female, and white male.
