# Supplementary material for: Trends in and disparities for acute myocardial infarction: an analysis of Medicare claims data from 1992 to 2010
Source: BMC Med. 2014 Oct 24;12:190. doi: 10.1186/s12916-014-0190-6 (PMC4212130; doi:10.1186/s12916-014-0190-6)
Supplement: Additional file 3: — Percentage of White and Black Men and Women who underwent percutaneous coronary intervention (PCI) within 30-day of admission for AMI. [file 12916_2014_190_MOESM3_ESM.pdf]

Additional file 3. Percentage of White and Black Men and Women who underwent percutaneous coronary intervention (PCI) within 30-day of admission for AMI

	BLACK FEMALE	BLACK MALE	WHITE FEMALE	WHITE MALE
**Number (%)**				
**Percent of all PCI in 30-day period that were performed during the index admission for initial AMI[2]**				
1992-1993	857 (61.5)	754 (60.7)	14567 (52.0)	19915 (54.2)
1994-1995	1241 (61.8)	1120 (63.4)	19519 (53.6)	26582 (56.5)
1996-1997	1600 (64.4)	1329 (62.7)	24381 (56.9)	31498 (58.9)
1998-1999	1841 (64.4)	1497 (64.1)	28156 (59.3)	35960 (61.2)
2000-2001	2330 (68.6)	2026 (67.1)	33527 (62.1)	42653 (64.4)
2002-2003	2916 (70.5)	2607 (71.8)	40308 (67.9)	51497 (70.0)
2004-2005	2972 (75.1)	2802 (77.8)	40402 (73.8)	52304 (75.8)
2007-2008	2999 (80.0)	2926 (82.1)	40387 (80.9)	54283 (82.7)
2009-2010	3315 (84.7)	3148 (84.4)	38285 (84.4)	53819 (85.8)
**Percent of all PCI in 30-day period that were performed after transfer to another hospital[2]**				
1992-1993	419 (30.1)	377 (30.3)	10967 (39.2)	13414 (36.5)
1994-1995	613 (30.5)	516 (29.2)	13971 (38.4)	16617 (35.3)
1996-1997	713 (28.7)	648 (30.6)	15327 (35.7)	17899 (33.5)
1998-1999	814 (28.4)	669 (28.6)	16011 (33.7)	18678 (31.8)
2000-2001	889 (26.2)	808 (26.8)	16971 (31.4)	19336 (29.2)
2002-2003	984 (23.8)	812 (22.4)	15575 (26.3)	17848 (24.2)
2004-2005	781 (19.7)	591 (16.4)	11365 (20.8)	12919 (18.7)
2007-2008	585 (15.6)	454 (12.7)	7079 (14.2)	8118 (12.4)
2009-2010	424 (10.8)	398 (10.7)	5182 (11.4)	6293 (10.0)
**Percent of all PCI in 30-day period that were performed during a different admission (but within 30-day of the onset of AMI)[2]**				
1992-1993	118 (8.5)	112 (9.0)	2479 (8.9)	3414 (9.3)
1994-1995	155 (7.7)	132 (7.5)	2916 (8.0)	3858 (8.2)
1996-1997	170 (6.9)	143 (6.8)	3177 (7.4)	4098 (7.6)
1998-1999	206 (7.2)	169 (7.2)	3339 (7.0)	4108 (7.0)
2000-2001	179 (5.3)	185 (6.1)	3483 (6.5)	4200 (6.5)
2002-2003	234 (5.7)	214 (5.9)	3448 (5.8)	4275 (5.8)
2004-2005	203 (5.1)	209 (5.8)	3009 (5.5)	3750 (5.4)
2007-2008	164 (4.4)	184 (5.2)	2472 (5.0)	3271 (5.0)
2009-2010	177 (4.5)	182 (4.9)	1908 (4.2)	2593 (4.1)

[1] The denominator is AMI patients in each strata, i.e., black female, black male, white female, and white male.

[2] The denominator is AMI patients who underwent PTCA in each strata, i.e., black female, black male, white female, and white male.
