# Supplementary material for: Trends in and disparities for acute myocardial infarction: an analysis of Medicare claims data from 1992 to 2010
Source: BMC Med. 2014 Oct 24;12:190. doi: 10.1186/s12916-014-0190-6 (PMC4212130; doi:10.1186/s12916-014-0190-6)
Supplement: Additional file 4: — Percentage of White and Black Men and Women who underwent Coronary Artery Bypass Graft (CABG) within 30-day after admission for AMI. [file 12916_2014_190_MOESM4_ESM.pdf]

Additional file 4. Percentage of White and Black Men and Women who underwent Coronary Artery Bypass Graft (CABG) within 30-day after admission for AMI

	BLACK FEMALE	BLACK MALE	WHITE FEMALE	WHITE MALE
**Percent of all CABG in 30-day period that were performed during the index admission for initial AMI[2]**				
1992-1993	519 (51.3)	435 (47.3)	7747 (40.7)	14152 (42.2)
1994-1995	624 (48.9)	647 (51.6)	9537 (41.4)	17294 (43.5)
1996-1997	713 (49.1)	729 (52.0)	10868 (43.5)	19127 (45.8)
1998-1999	766 (51.7)	703 (52.9)	10541 (46.6)	18400 (48.7)
2000-2001	871 (54.3)	770 (52.7)	10617 (49.2)	18859 (51.4)
2002-2003	913 (58.2)	905 (58.5)	10726 (54.6)	19471 (56.9)
2004-2005	704 (60.7)	787 (63.0)	8822 (60.6)	16693 (63.4)
2007-2008	604 (64.5)	697 (65.5)	7369 (66.2)	14595 (68.7)
2009-2010	565 (68.1)	648 (67.9)	5883 (66.8)	12332 (69.4)
**Percent of all CABG in 30-day period that were performed after transfer to another hospital[2]**				
1992-1993	382 (37.8)	353 (38.4)	9065 (47.6)	14901 (44.5)
1994-1995	500 (39.2)	461 (36.8)	10834 (47.0)	17742 (44.7)
1996-1997	577 (39.7)	516 (36.8)	11498 (46.0)	18039 (43.2)
1998-1999	565 (38.1)	491 (37.0)	9790 (43.3)	15647 (41.4)
2000-2001	579 (36.1)	530 (36.3)	8905 (41.3)	14371 (39.2)
2002-2003	495 (31.6)	520 (33.6)	7364 (37.5)	11900 (34.8)
2004-2005	364 (31.4)	363 (29.0)	4671 (32.1)	7636 (29.0)
2007-2008	242 (25.8)	272 (25.6)	2956 (26.6)	5107 (24.0)
2009-2010	194 (23.4)	218 (22.9)	2248 (25.5)	4072 (22.9)
**Percent of all CABG performed in 30-day period that were during a different admission (but within 30-day of the onset of AMI)[2]**				
1992-1993	110 (10.9)	131 (14.3)	2246 (11.8)	4455 (13.3)
1994-1995	153 (12.0)	146 (11.6)	2676 (11.6)	4703 (11.8)
1996-1997	163 (11.2)	157 (11.2)	2640 (10.6)	4623 (11.1)
1998-1999	152 (10.3)	135 (10.2)	2295 (10.1)	3749 (9.9)
2000-2001	153 (9.5)	161 (11.0)	2045 (9.5)	3460 (9.4)
2002-2003	161 (10.3)	122 (7.9)	1564 (8.0)	2855 (8.3)
2004-2005	92 (7.9)	100 (8.0)	1067 (7.3)	2013 (7.6)
2007-2008	91 (9.7)	95 (8.9)	800 (7.2)	1547 (7.3)
2009-2010	71 (8.6)	88 (9.2)	675 (7.7)	1369 (7.7)

[1] The denominator is AMI patients in each strata, i.e., black female, black male, white female, and white male.

[2] The denominator is AMI patients who underwent CABG in each strata, i.e., black female, black male, white female, and white male.
