# Supplementary material for: Trends in and disparities for acute myocardial infarction: an analysis of Medicare claims data from 1992 to 2010
Source: BMC Med. 2014 Oct 24;12:190. doi: 10.1186/s12916-014-0190-6 (PMC4212130; doi:10.1186/s12916-014-0190-6)

Additional file 6. Procedural Utilization During the Index Admission for initial AMI Adjusted for Patient Age and Comorbidities, and Clustering of Patients within Hospitals

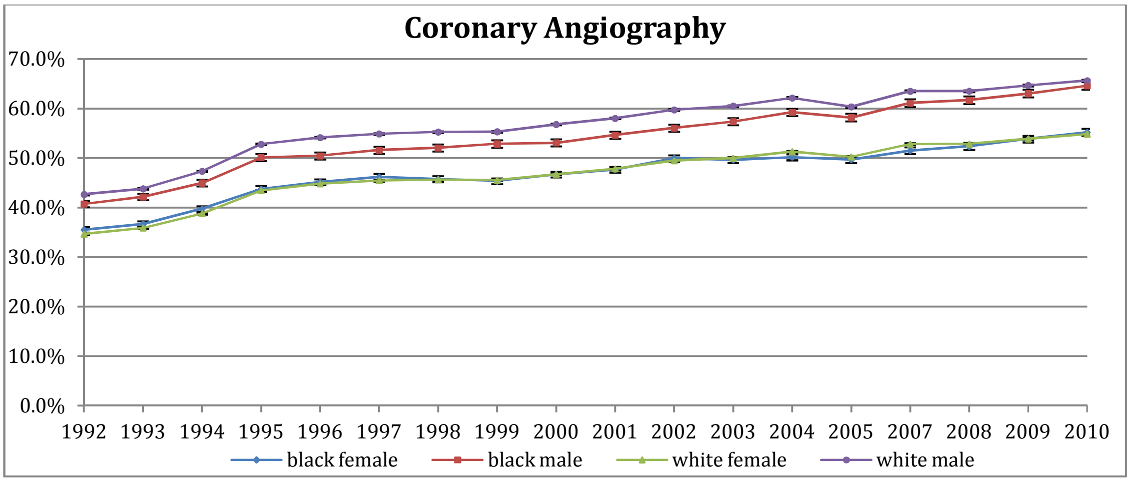

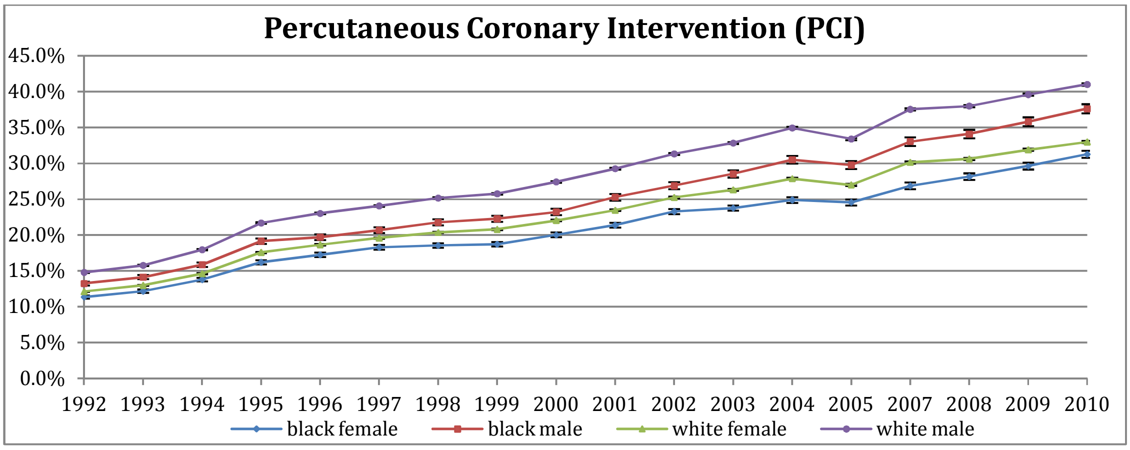

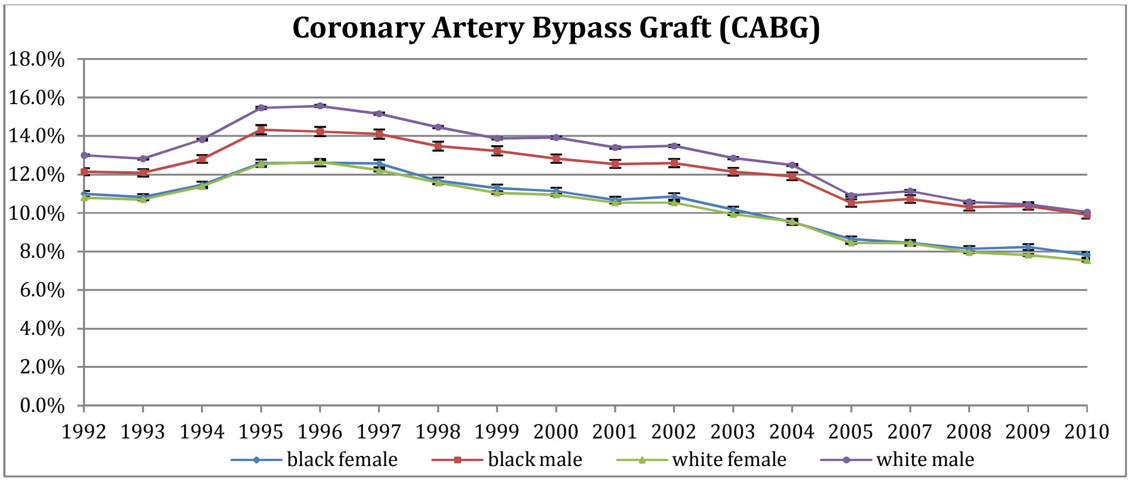

Supplement: Additional file 6: — Procedural Utilization During the Index Admission for initial AMI Adjusted for Patient Age and Comorbidities, and Clustering of Patients within Hospitals. [file 12916_2014_190_MOESM6_ESM.pdf]
